# Examining the intersection of cognitive & physical function in the Brain Networks and Mobility Function (BNET) Study

**DOI:** 10.1093/geroni/igaa057.3305

**Published:** 2020-12-16

**Authors:** Elizabeth Handing, Michael Miller, Haiying Chen, Laura Baker, Stephen Kritchevsky

**Affiliations:** Wake Forest School of Medicine, Winston-Salem, North Carolina, United States

## Abstract

Cognitive function and physical function are associated however less is known about task complexity and how individual tasks relate to one another. This project seeks to describe the relationship between cognition and physical function measures across 22 tasks ranging in task complexity and difficulty. Data are from the baseline visits of a new longitudinal study, Brain Networks and Mobility Function (B-NET) Study, mean age: 76.0±4.2 years; 55% women, and 90% Caucasian. We hypothesize there would be a set of “complex” tasks that would intersect both cognitive and physical function abilities such as the Four Square Step Test or Dual Task. We conducted principal components analysis on data from the first 110 participants to describe what factors could be identified across cognition and physical function measures. Seven factors, explaining 73% of the variability, were identified: 1) a complex physical function (postural sway on foam, expanded Short Physical Performance Battery, 400 meter walk, Four Square Step Test, Dual Task), 2) physical strength (grip strength and leg press), 3) visual recall (Brief Visuospatial Memory Test- immediate and delayed), 4) Craft story recall (immediate and delayed), 5) global cognition & fluency (MoCA, category and word fluency) 6) auditory recall (Auditory Verbal Learning Test- immediate and delayed), and 7) executive function (Trail Making Test A & B). We did not identify factors that intersected both physical and cognitive tasks. These results may help to inform measurement selection in future studies that seek to evaluate components of function among older adults.

